# Comparative Analysis of the Wear of NC11LV and Hardox 600 Steel Used in Tools for Extrusion of Clay Strands in the Process of Producing Ceramic Roof Tiles

**DOI:** 10.3390/ma16010293

**Published:** 2022-12-28

**Authors:** Marek Hawryluk, Marzena M. Lachowicz, Jan Marzec, Kamila Nowak, Maciej Suliga

**Affiliations:** 1 Department of Metal Forming, Welding and Metrology, Faculty of Mechanical Engineering, Wroclaw University of Science and Technology, Lukasiewicza Street 5, 50-370 Wroclaw, Poland; 2 Faculty of Chemistry, Wroclaw University of Science and Technology, Norwida Street 4/6, 50-373 Wrocław, Poland; 3 Faculty of Production Engineering and Materials Technology, Czestochowa University of Technology, Al. Armii Krajowej 19, 42-201 Częstochowa, Poland

**Keywords:** tool life, roof tiles, tribological wear, ball-on-disc test, increased wear resistance, microstructure

## Abstract

This article presents the results of a comparative analysis performed with respect to the wear of tools used for the extrusion of a clay strand (for ceramic roof tile) made from two materials: steel NC11LV and steel Hardox 600. The aim of the studies was to determine the causes and mechanisms of wear as well as to evaluate the possibility of choosing the optimal material, mostly in respect to its resistance to intensive wear as well as an increase in the operation time. The results of the conducted investigations included: an analysis of the technology, thermovision measurements of the forming process, a macroscopic analysis combined with 3D scanning of the worn tools, ball-on-disc tests of the sliding wear resistance and hardness measurements. The obtained results demonstrated that the tools made of steel NC11LV were much less worn than those made of steel Hardox 600, as the operation time for the NC11LV steel tools was almost three times longer. The results of the ball-on-disc tests showed a similar manner of wear for both materials (with the working temperature of about 50 °C). The higher durability of the tools made from steel NC11LV can be an effect of a slightly lower coefficient of friction in the initial period of operation as well as the presence of hard carbides, which means increased hardness and thus also higher wear resistance at working temperatures.

## 1. Introduction

At present, the most commonly applied roofing materials include: steel roof tiles, bituminous roof tiles, concrete and ceramic roof tiles, etc. [1,2]. Due to the most advantageous parameters of all the roofing materials and the quality–price ratio, the most optimal variant is still represented by ceramic roof tiles, which characterize, over a very long operation time, a natural origin and the possibility of being placed on roofs with a minimal roof pitch, etc. A ceramic tile is made from natural resources and constitutes an ecological type of roofing. It is burned from clay coated with liquid clay, with an additive of minerals or metal oxides. Such high universality of this type of roof tiles has an effect on the broadly understood development of the ceramic industry [3]. One should note the fact that, at the time of a global crisis of resources and energy price increase, the main focus of the development of the ceramic industry in the area of roof tile production is environmental protection [4] as well as recycling of post-production waste [5]. One of the aspects of environmental protection is the reduction of wear and friction, which are estimated to be responsible for 2.7% of global CO_2_ emission [6]. Another direction of development is the search for new, alternative materials for ceramic roof tiles, optimization of the materials applied in the production of machine elements used to produce roof tiles and continuous development of the roof tile production technology [7]. This is connected with the more and more rigorous environmental requirements and the constantly increasing efficiency and competitiveness of production. It should be clearly emphasized that the key elements of the mechanisms and devices on the roof tile production lines, which are in direct contact with the extruded material, have to be especially resistant to abrasive wear occurring due to contact with the extruded strand of clay. For the extrusion of a strand of a material whose main component is clay and quartz sand, horizontal multi-band extrusion machines are used. At the end of the latter, there is usually a worm gear tipped with a so-called orifice, equipped with a set of two or more tools (bits) and forming the final shape of the exiting strand [8,9,10]. Materials used for working machine parts should be selected with respect to the processed production mass. The basic parameters of the mass include: amount of clay, amount of sand, grain size and moisture [11]. The extruders process the mass with a moisture content of 18–25% and under pressure of 3–15 MPa, which cause the working surfaces of the plates to be exposed to very high stresses. This results in their intensive abrasive wear and temperature increase as well as the occurrence of other destructive mechanisms and phenomena [12]. The operation of these types of tools is affected by many, often opposing, factors and physico-chemical phenomena, which makes the analysis of their wear difficult and complex. It is also the case of shaping tools in similar manufacturing processes, e.g., in forging processes or punches in extrusion processes (variable pressures and temperature gradients, large friction path, etc.) [13,14,15]. According to literature data, machines, devices and tools are subject to surface layer wear processes, in which 60% of elements are worn in the abrasion process, 10–15% as a result of adhesion, 5–7% as an effect of erosion and 2% are oxidized. Based on the literature review, different types of abrasive wear may occur in industrial processes. First of all, the hardness of the particles and their structure, grain size and shape determine the type of abrasive wear. The hard particles of the abrasive can deform the metal elastically and plastically (causing furrowing) or micro-cut the surfaces during the movement of the abrasive tangentially to the surface of the element [16]. When the surface of the tool has already been strengthened as a result of the previous plastic deformation of the abrasive and the strengthening mechanism is no longer observed, the surface is chipped [17]. As a result of the abrasive particles moving relative to each other at high speeds, the heat generated from friction can significantly affect the abrasive wear and even cause changes in the internal structure of the material. This can be manifested by a local reduction in mechanical properties and, as a consequence, lead to premature wear of the element. Increasing the durability of tools subjected to intensive abrasion is possible through the use of special materials resistant to abrasive wear. Among the materials assigned, e.g., for tools used to squeeze a strand of clay, several groups can be distinguished: wear-resistant steels (with commercial names such as Hardox, RAEXs, XAAR), boron steels, manganese steels (Hadfield) and heat-treated cold work tool steels (NC10, NC11, NC11LV). In regard to the relatively expensive wear-resistant steels, for economic reasons, they are most often used as a replaceable working element, which is subject to relatively higher intensive wear and is combined with a wear-free element with lower abrasion resistance (cheaper). [18,19]. In the relatively expensive boron steels, the main alloying element, boron, is dissolved in the austenite, which means that, in a typical hardening process, a bainite structure with fine grains and high hardness can be obtained. Manganese steels (about 12–13% manganese, additional to 1% carbon) are characterized by a high tendency for work hardening, which occurs under conditions of high surface pressure. At the same time, a big problem of these types of steel (Hadfield steel) is their machinability [20]. Therefore, a certain alternative, especially in terms of relating the required mechanical properties to the price, is the aforementioned cold work tool steels. Steels used for cold work (high-alloy) with high abrasion resistance include NC11LV, NC11 and NCWV steels, owing to their high chromium content (about 11%) [21,22]. According to the European norms, steel NC11LV corresponds to the grade X153CrMoV12 (Steel 1.2379). An equivalent of this steel in the ASTM AISI norms is grade D2. In this type of steel, mainly multicomponent carbides are formed. For example, steel NC11LV may contain carbides M_7_C_3_ or M_23_C_6_, where M means metal, which significantly increase the hardness and wear resistance of elements made of these materials. An intermediate transition metal carbide structure is more complex than interstitial carbides. In the case of steel NC11LV, the austenitizing temperature is very important and should not exceed 1050 °C if the highest hardness is to be obtained. In addition, other ways to increase the hardness include the use of various thermo-chemical treatments. The high hardness of this steel is mainly due to numerous precipitations of very hard secondary and primary carbides [23]. An additional advantage of steel NC11LV is the fact that it also strengthens as a result of deformation, which makes it well suited as a tool for pressing a strip of clay onto ceramic roof tiles [24]. In the available literature, one can find many studies and analyses related to the issue of wear as well as the selection of various material solutions for increasing the durability of tools operating under high friction conditions [25]. On the other hand, few literature studies can be found that relate strictly to tools used in the processes of extruding a strip of clay onto roof tiles [26]. Of course, apart from material solutions, an increase in the operation time of tools used in the clay strand extruding processes can be achieved through optimization of the technological parameters as well as special tool construction solutions [27,28]. For this reason, conducting advanced studies in this area aimed at an analysis of premature wear of tools exposed to intensive abrasive wear and also proposing ways and methods of their improvement is fully justified, as it constitutes a problem both in the scientific and economical aspect. In view of the intensive development of information technology, more and more often, mathematical solutions are applied, which support the designing and numerical modelling of the strand extruding processes with the use of CFD (Computational Fluid Dynamics). There are currently many solutions on the market which use CFD to solve issues of this type, of which the most important ones include: FLUENT, STAR-CD, CFX, FLOW 3D, PHOENICS, FEMLAB and Ansys-FLUENT, where most of these programs are based on the FVM technique (Finite Element Method) [FEM] [29,30,31,32]. At present, advanced studies are being performed which aim at determining the tribological wear resistance of materials used for machine elements. A commonly applied method of determining the tribological properties of materials is the ball-on-disc test. This scientific method belongs to the most frequently selected ones, as it can represent the operation conditions present in various industrial branches, e.g., the extractive industry, which, in terms of wear and friction, is very similar to the ceramic industry [33]. For this reason, the occurring material wear is significantly affected by the assumed parameters of the ball-on-disc test, which include: load, speed, temperature and time [34]. The results of the ball-on-disc tests show that there are many alternatives for abrasion-resisting Hardox steels. One of such alternatives is the high-strength low-alloy (HSLA) steel in the QT state (quenched and tempered). This shows that steels with similar hardness but a more fine-grained structure through controlled thermomechanical processing and higher yield point exhibit better tribological properties than steel Hardox [35]. An improvement in these properties can be ensured by the application of additional thermo-chemical treatment, such as nitriding or carburizing, which makes it possible to reduce the coefficient of friction up to a few times [36]. The results of the performed ball-on-disc tests also demonstrate that an important role in the determination of the abrasive properties is played by the applied thermal treatment as well as the formed microstructure of steel. In the case of tool steels, the presence of primary carbide precipitates formed during solidification, and secondary carbides formed from the martensitic matrix during tempering, is very important. Their size, shape and distribution play an important role in the friction process and the following material wear [37,38]. This study conducts a complex comparative analysis of the wear of the selected tools, so-called bits, used to form strands for roof tiles made from two different materials: steel Hardox 600 and an alternative material, steel NC11LV, in order to determine the causes and mechanisms of their wear.

The main scientific aim is a comparison of the wear of the tools used in the extrusion of a clay strand from steel Hardox and steel NC11LV through an analysis of their wear in the production process as well as by means of ball-on-disc tests. This will enable a determination of the relationship between the coefficient of friction and the horizontal material wear and should facilitate selection of the optimal tool material solution.

## 2. Materials and Methods

**Figure 1:**
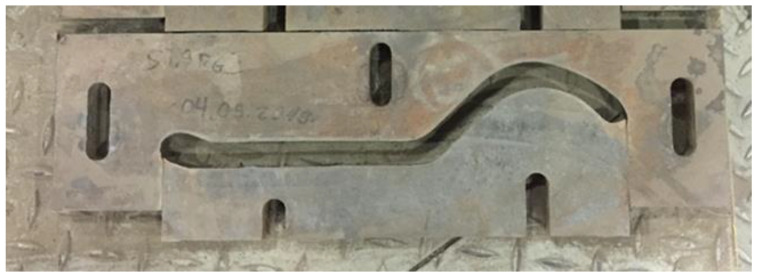
Tool forming a clay strand (bit) divided into the upper and lower element.

Figure 1 shows a photo of tools (bits) used to form a clay strand in the process of ceramic roof tile production. The bits are especially problematic elements due to the intensive abrasive wear of their working surfaces, which are in contact with the strand of the formed clay. The materials used for tools assigned for such applications are characterized by high abrasive resistance, also at elevated temperatures.

In the analyzed process, the tools are made of metal plate with a thickness of 30 mm from steel Hardox 600 (steel needs no additional thermal treatment), which is in direct contact with the processed material. For the preparation of the second set of tools, steel NC11LV (X153CrMoV12, Steel 1.2379, AISI D2) was selected, which has similar properties to steel Hardox. The classical heat treatment of NC11LV steel consists of austenitizing at 1020 °C, air cooling and then tempering at 180 °C. According to the PN-EN ISO 4957 standard, the hardness should be at least 61 HRC. Both tools were produced in an abrasive cutting water-jet machining process.

The chemical composition of the tool materials determined with the use of the glow-discharge optical emission spectroscopy (GD OES) method on the Leco GDS 500A analyzer (Leco Corporation, St. Joseph, MI, USA) is shown in Table 1.

In order to perform a complex analysis, the following tests were carried out:

- Analysis of the process and operation time. The tool temperatures were measured with the use of a pyrometer (pyrometerTesto 845, Testo Poland, Pruszkow, Poland) and a thermovision camera (FLIR T530, FLIR Systems, Inc. Wilsonville, OR, USA). The times of the consecutive operations (strand extrusion speed) were determined by means of a high-speed camera (Casio Exilim Pro EX-F1, Casio, Tokio, Japan) capable of recording over 1000 frames per second.
- Macroscopic examinations were carried out in order to select samples and areas with the highest wear for further examination. The results of visual observations were recorded using a Canon EOS 50D camera (Canon Inc., Tokio, Japan).
- For the evaluation of the working surface’s geometry, a measuring arm ROMER Absolute ARM 7520si integrated with an RS3 scanner, Hexagon Manufacturing Intelligence, Aarau, Switzerland) together with the Polyworks software (2015, Hexagon Manufacturing Intelligence, Aarau, Switzerland) were used, which made it possible to perform 3D scanning in the Real Time Quality Meshing technology.
- A ball-on-disc sliding wear test was performed to estimate the tribological properties under the conditions of dry sliding friction for the wear couple: ball Al_2_O_3_-steel disc Hardox 600 and NC11LV (samples cut out of the tools), load 10 N, distance 300 m, by sliding speed 0.1 m/s.
- Microstructural studies were performed with the use of a metallographic microscope Leica, model DM6000M (Leica Microsystems, Wetzlar, Hesse, Germany) and a scanning electron microscope Phenom Pro X. A 4% solution (Thermo Fisher Scientific, Waltham, MA, USA) of pirkynic acid in ethyl alcohol was used for the etching of the metallographic specimens.
- In order to determine the hardness distribution, hardness measurements were carried out with the use of the Vickers method by means of a Leco AMH55 hardness tester (Leco Corporation, St. Joseph, MI, USA). Considering that, in the engineering practice, the Rockwell hardness is mainly used for hardened steels, a decision was made to convert the Vickers hardness (HV1) to the Rockwell hardness. On this basis, graphs of the hardness’ dependence on the distance from the working surface of the tool were determined. The tests were carried out on metallographic specimens prepared on the cross-section of the tools.

## 3. Results

### 3.1. Analysis of Extrusion under Industrial Conditions

**Figure 2:**
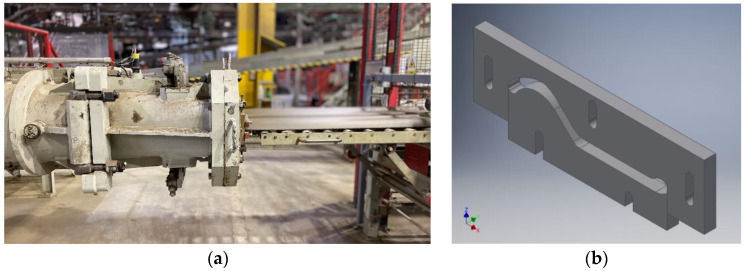
Process of forming a clay strand: (**a**) extruding the strand on a pug mill, (**b**) a 3D model of the tool shaping the strand (bit).

The process of producing ceramic roof tiles consists of five main stages, which include: raw material recovery, body preparation, semi-finished product formation, drying and final product firing. Among these processes, the forming is especially important, which can be additionally divided into a few sub-processes, such as: body homogenization in a two-shank mixer, raw material venting in a vacuum chamber, strand forming and cutting into the specific size and creating the final shape of the roof tile on a punch press. The process of forming the strand is presented in Figure 2. The homogenized body, vented in a pug mill, is extruded under pressure in the head (Figure 2a) and is formed into a strand by means of forming tools (bits), whose 3D model has been shown in Figure 2b.

**Figure 3:**
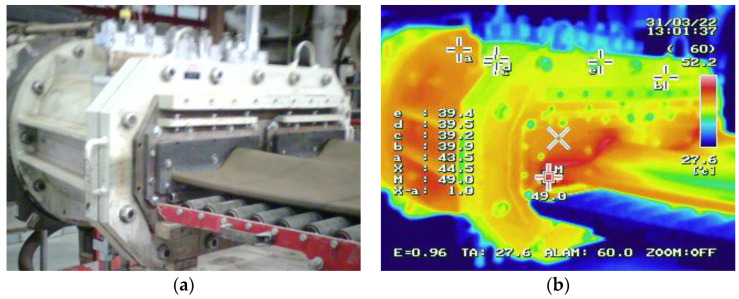
View of: (**a**) a photograph of the strand extruded through a pug mill, (**b**) a thermovision diagram of the clay strand and tool set.

The tools form the strand by providing it with the proper shape and thickness. Special attention should be paid to the degree and type of wear, which can have an effect on the quality of the end product. The process of extruding the strand takes place under the pressure of about 10 MPa, which results in intensive abrasion and temperature increase in the clay strand–tool contact in the scope of 44–50 °C (Figure 3), and the speed of the extruded strand mixture equals about 0.4 m/s.

The increased temperature in the area of the tool’s contact with the extruded material is caused by the occurrence of intensive friction in this area. Such a temperature value on the tools should positively affect the impact strength and thus prolong the operation time of the tool in such a process.

### 3.2. Visual Analysis

**Figure 4:**
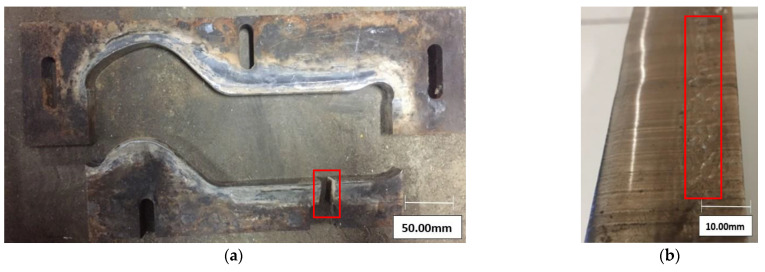

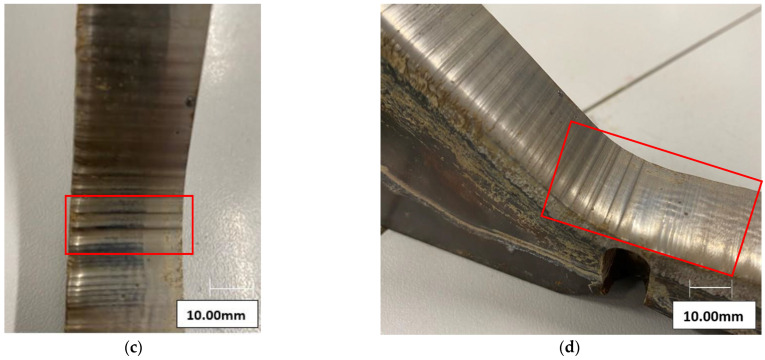
The general view of bits made of steel Hardox forming the clay strand based on visual inspection: (**a**) cracks, (**b**) traces of impressions, (**c**) longitudinal scratches, (**d**) scratches and cavities in the transverse direction.

The performed complex macroscopic analysis of tools made of both analyzed materials demonstrated that their wear was similar. Figure 4 shows photographs of the most frequent flaws and typical performance wear. We can observe shallow scratches both in the direction of the extruded strand (Figure 4a) and in the perpendicular direction (Figure 4d). Traces of impressions coming from harder fractions in the clay are also observed (Figure 4b) as well as scratches and cavities, possibly pointing to the occurrence of plastic deformations (Figure 4c).

The results of the macroscopic analysis of the tools made from both materials are the most commonly observed flaws and defects, caused by the operation conditions, especially the presence of hard particles (coming from the milled remainders of the burned roof tiles being one of the strand components). A result of their operation is the formation of numerous scratches on the internal surface of the bit. This can lead to their representation on the strand surface and further constitute a defect of the product, i.e., roof tile.

The conducted analysis of the operation time of the selected tool sets demonstrated that the tools made of steel NC11LV had worked over about 900 operation hours, whereas the tools made from steel Hardox 600 had worked over about 400 operation hours.

### 3.3. Analysis of Geometrical Changes with 3D Scanning

**Figure 5:**
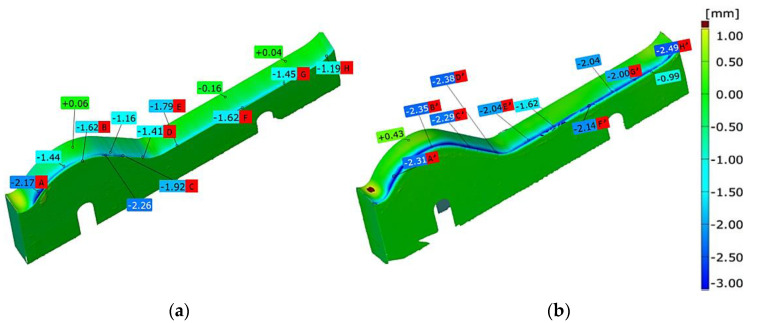
Results of 3D scanning: (**a**) Hardox 600 after 400 h of work and (**b**) NC11LV after 900 h of work.

Through the use of 3D scanning, measurements, followed by an analysis of the geometrical changes of the lower tools, were performed, which demonstrated their wear. Figure 5 shows the scanning results for the lower tools made of both materials, which are usually non-adjustable. The upper tools can be adjusted during the process (shifted downwards on a slotted hole, in such a way so that it is possible to maintain a specific strand thickness). In turn, in the case of a profile change, the upper tools are completely removed from the process.

In the analysis of the obtained results for the lower tool made of steel Hardox 600 (Figure 5a), we can state that the material loss is practically uniform on the whole forming line, yet greater on the input side, i.e., the nozzle. In turn, for the bit made from steel NC11LV (Figure 5b), the wear is lower, and the biggest material loss is in the areas of intensive abrasive wear, that is, in the areas responsible for the shaping of the so-called wave. In this zone, the wear reaches over 2 mm. In the remaining section of the bit, the highest wear is still visible on the edge, yet it oscillates between 1.5 mm to almost 2 mm. For the tools made from steel Hardox, the wear mechanism of the bit is similar but much more intensive. The biggest material loss takes place on the whole length of the edge on the side of the pug mill and, practically on the whole length of this edge, the wear reaches over 2 mm, with the maximum of 2.5 mm on the tip of the edge.

### 3.4. Hardness Measurements

**Figure 6:**
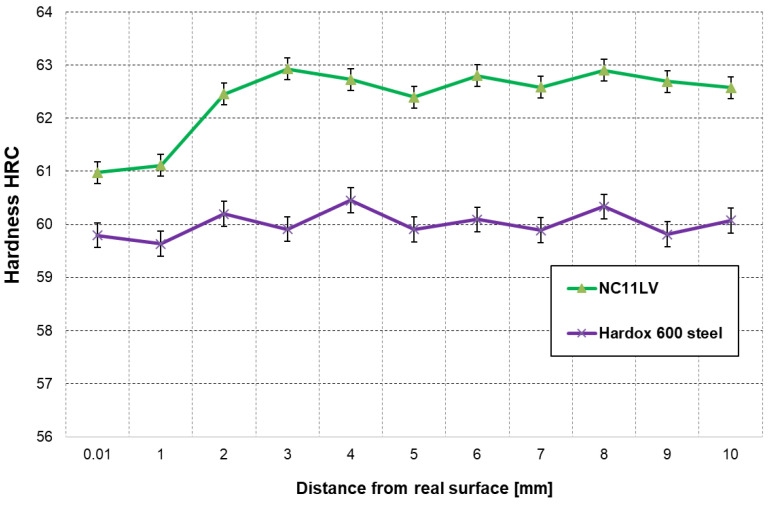
Hardness profiles at a distance from the working surface for tool samples made of steel NC11LV and Hardox 600.

Additionally, in order to evaluate the examined materials, a hardness measurement was performed for the lower tools made from both materials in the actual area of contact with the strand towards the inside of the material (Figure 6).

For steel NC11LV, the material hardness is at the level of about 61 HRC. A slight decrease in hardness in the near-surface area was observed as a result of high normal pressures coming from the abrasion and the resulting tempering. Towards the inside of the material, the hardness stabilizes at the level of about 62–63 HRC. In turn, for steel Hardox 600, the hardness is stabilized in the whole depth and equals about 60 HRC.

### 3.5. Ball-on-Disc Tests

**Figure 7:**
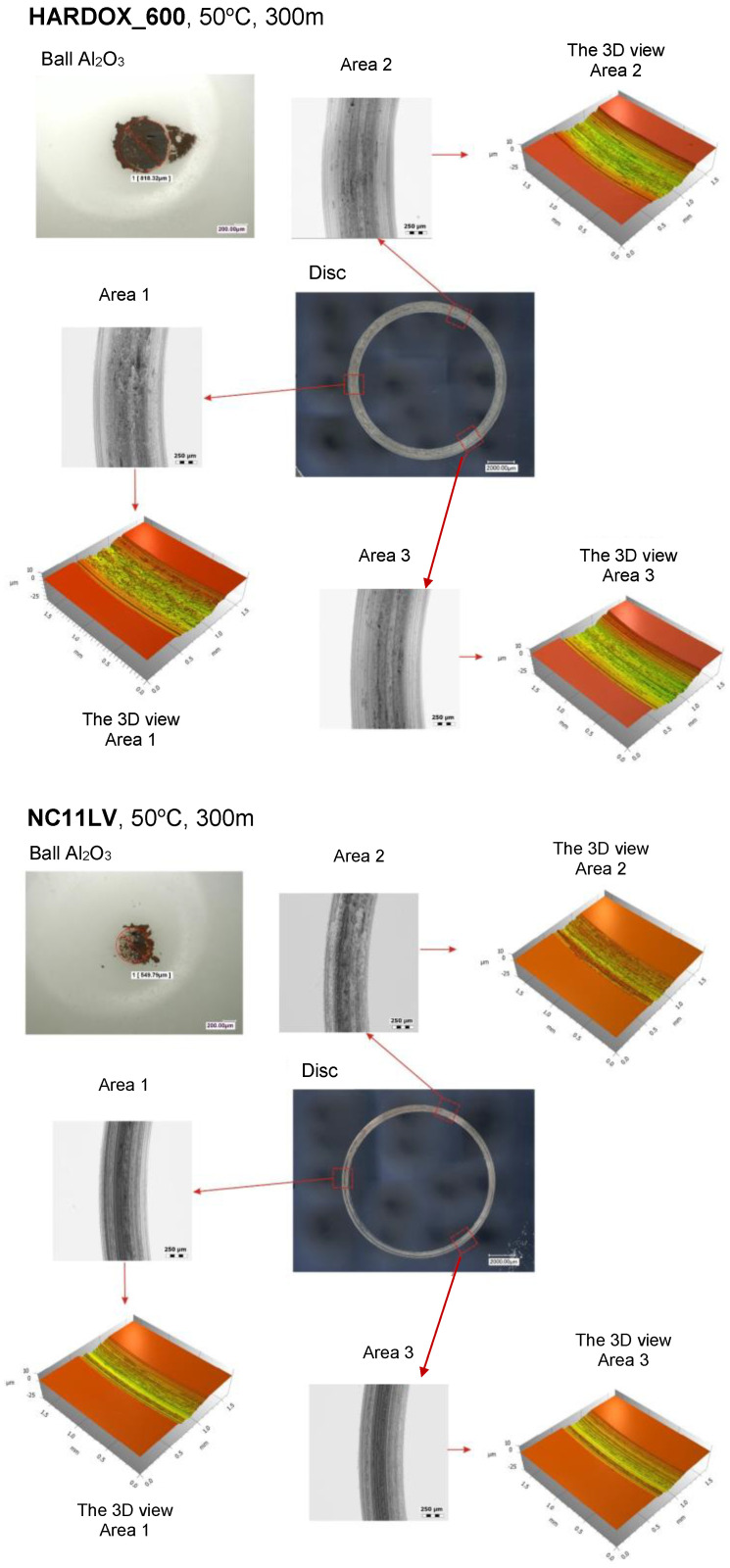
Results of ball-on-disc tests for the analyzed materials.

During the ball-on-disc tests, the abrasion track was determined, which enabled the determination of wear of the analyzed materials: Hardox and NC11LV, with a ball Al_2_O_3_ at 50 °C, as, on the external tool surfaces, a temperature of about 40 °C was observed during the thermovision measurements, and it was assumed that, in direct contact, it could be higher. Figure 7 shows the test results for steel Hardox on the length of 300 m together with 3 magnified areas of wear traces as well as the results of the measured depth and width of the tracks. In the analysis of the obtained investigation results, we can see that the wear traces for steel Hardox 600 are much deeper and wider. The mean depths for the 3 selected areas in the case of this steel equal about 17.7 micrometers, whereas for steel NC11LV, they are about 6.6 micrometers. We can also observe a slightly different character of wear, i.e., it is more uniform and wider for steel Hardox 600, while in the case of steel NC11LV, we can notice certain irregularities, possibly suggesting the presence of hard carbide bands. Additionally, in the case of the results for steel Hardox, we can see that, despite its deeper track of wear, the latter is characteristic of a material which has a more stable microstructure trace, as it reflects the shape of the ball.

**Figure 8:**
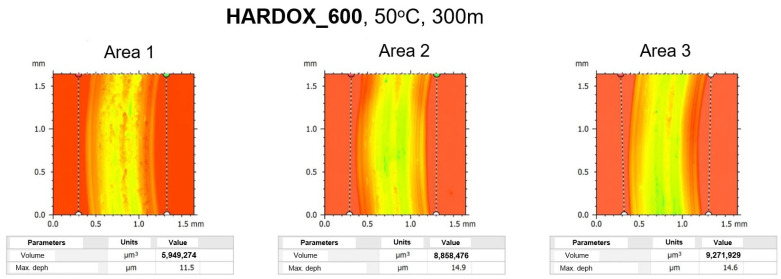

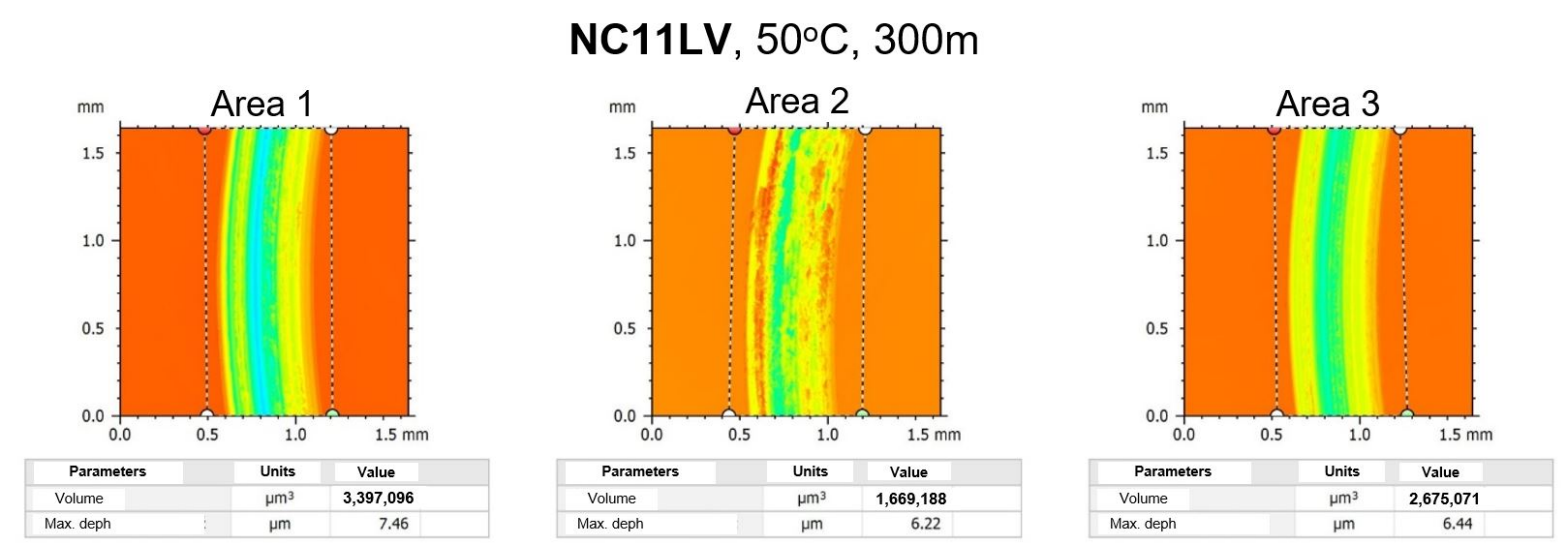
Detailed characteristics of material loss for the analyzed materials.

The structure’s stability is also confirmed by the fact that the wear in the three selected areas for steel Hardox 600 is very similar, contrary to the case of steel NC11LV, where, for each selected area, the character of wear is slightly different. Figure 8 presents detailed characteristics for the analyzed materials for the three areas selected from the whole test track. As we can see, in the case of steel Hardox, the depths of wear are three times higher than for steel NC11LV.

Table 2 presents the selected mean parameter from the ball-on-disc tests for the examined materials.

**Figure 9:**
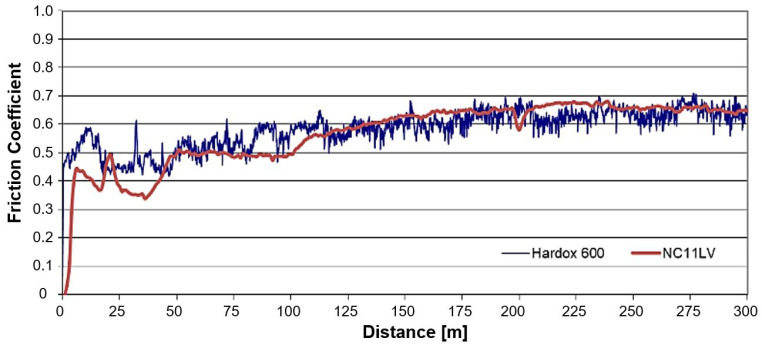
Coefficients of friction in a ball-on-disc trial for both analyzed materials.

Within the ball-on-disc tests, the values of friction coefficients from the trial for the whole 300 m measurement track were also determined (Figure 9).

In the analysis of the obtained results, there are no visible differences in the courses of the coefficients of friction. Initially, slightly lower values are recorded for both materials, whereas after about 70 m, we can see a slight increase in the coefficients of friction, which can be explained by an increase in oxidation of the friction track’s surface in the samples. The mean value in both cases oscillates around 0.47.

### 3.6. State of the Sample Surface after Ball-on-Disc Tests for the Analyzed Materials

**Figure 10:**
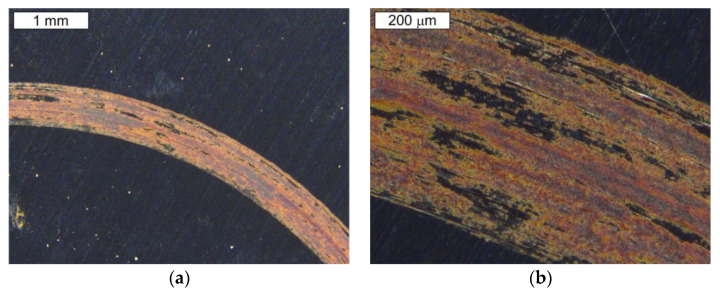
View of: (**a**) a stereoscopic image of the friction face for steel Hardox 600 and (**b**) a magnified fragment from the area in Figure 10a. Stereoscopic microscopy.

A top view of the friction faces for samples made of steel Hardox 600 in a stereoscopic image has been shown in Figure 10. There are visible surface changes covering their entire friction track as well as clear surface scratches. A microscopic SEM image obtained from a selected friction area is presented in Figure 10. Based on the obtained test results, it was established that the fundamental process occurring in the friction couple was surface oxidation, which led to the presence of oxides in the friction area. The matte areas visible in the image are responsible for the occurrence of oxides. The occurrence of numerous scratches on the surface was observed. A result of tribooxidation will be the generation of oxides on the side surfaces of elements made of this material, which are applied in tribological couples.

**Figure 11:**
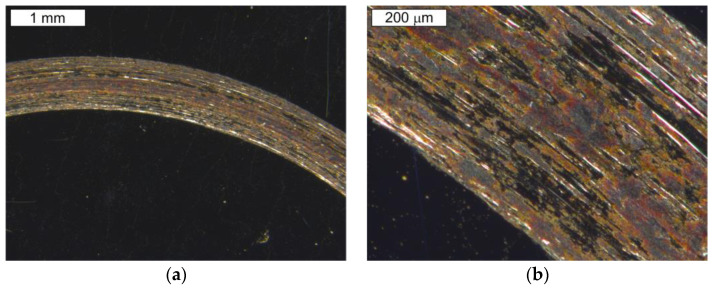
Photographs of: (**a**) a stereoscopic image of the friction face for steel NC11LV and (**b**) a magnified fragment of the area from Figure 11a. Stereoscopic microscopy.

A general view of the friction face for samples made of steel NC11LV in a stereoscopic image is shown in Figure 11. The character of the formed changes is similar to those in the case of steel Hardox. However, the share of oxidized areas is smaller. There are visible surface features covering the whole friction track as well as clear surface scratches. A microscopic SEM image obtained from a selected area has been presented in Figure 11b.

**Figure 12:**
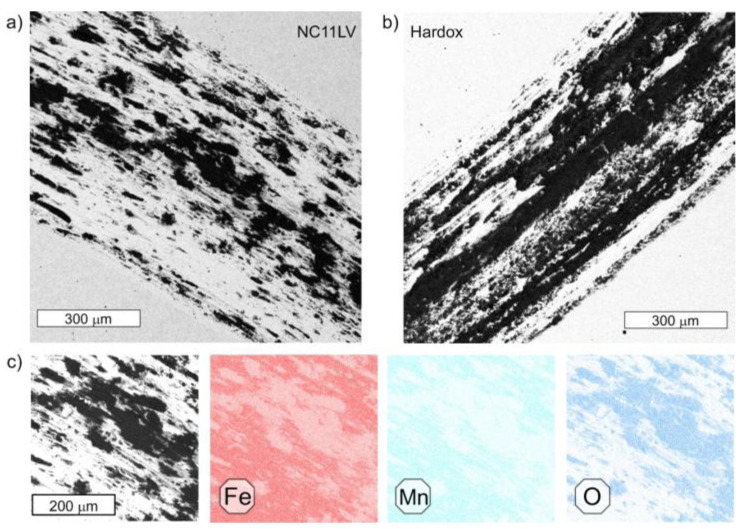
Microscopic SEM image: (**a**) obtained from the friction area for steel NC11LV and (**b**) obtained from the friction area for steel Hardox 600. (**c**) An enlarged fragment of the area in Figure 12a with the distribution of iron, manganese and oxygen. SEM/EDX.

In order to confirm the formation of oxides on the friction faces, the element distributions in this area were determined, confirming the presence of oxides, which points to the occurrence of tribooxidation. An exemplary microscopic image together with the obtained element distribution for NC11LV steel is shown in Figure 12. The dark areas in the microscopic image point to the oxide zone. The distribution of elements for Hardox 600 steel was analogical to that obtained for NC11LV steel.

In both the stereoscopic images (Figure 10 and Figure 11) and during the microscopic SEM observations, a lower tendency for surface oxidation was observed in the case of steel NC11LV than steel Hardox. This effect should be related to the significant difference between both steels in the chromium content, which, for steel NC11LV, equals on average of 12%. A part of the chromium is bound in the carbides, but the rest is also dissolved in the matrix. On the one hand, this ensures an increased oxidation resistance of the steel and, on the other hand, together with the high carbon content, it provides the possibility of the formation of numerous carbides, which ensure its improved hardness.

### 3.7. Microstructure Examinations

**Figure 13:**
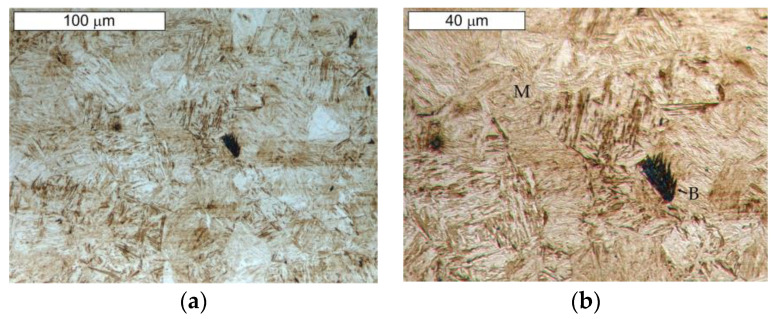
View of (**a**) the microstructure of steel Hardox and (**b**) a magnified fragment of the area in Figure 13a (M—martensite, B—bainite). Light microscopy, etched.

**Figure 14:**
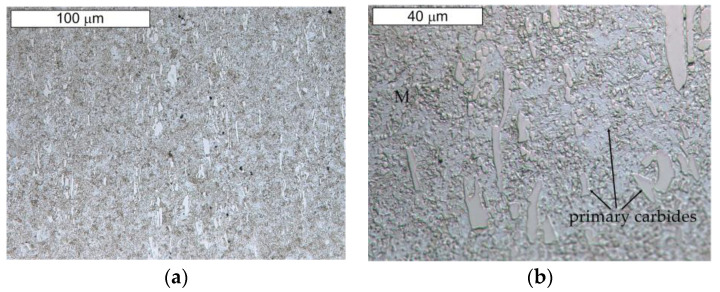
View of (**a**) the microstructure of steel NC11LV and (**b**) a magnified fragment of the area in Figure 14a (M—martensite). Light microscopy, etched.

Figure 13 and Figure 14 present the microstructure of the examined materials. In the case of steel Hardox, a martensitic–bainitic structure with single upper (feather) bainite was observed. The presence of bainite proves the relatively low hardenability of Hardox 600 steel, as well as the occurrence of microsegregation of the chemical composition. For steel NC11LV, the matrix was constituted by tempered martensite without diffusion-controlled structural components. On its background, bands of primary carbide precipitations were observed. The carbide bands were oriented perpendicular to the examined surface. The presence of primary carbides should translate into the intensity of wear of the tested materials.

### 3.8. Microscopic Tests on the Cross-Section

#### 3.8.1. Steel Hardox 600

**Figure 15:**
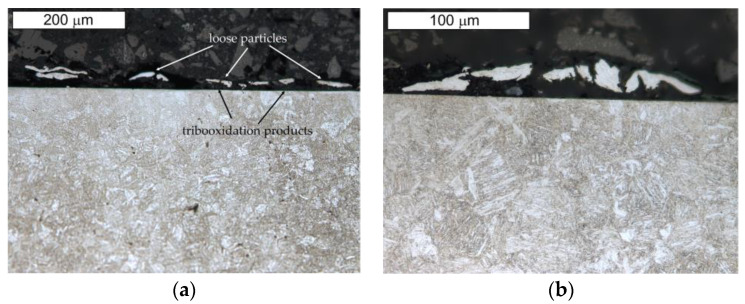
View of (**a**) the microstructure of steel Hardox in the surface area and (**b**) a magnified fragment of the area in Figure 15a. Light microscopy, etched.

Additionally, the microstructural tests of the analyzed samples of steel Hardox performed after the ball-on-disc tests confirmed the microscopic observations on the cross-sections of the examined samples. The presence of loose particles on the surface and of the related oxidation products was established (Figure 15).

**Figure 16:**
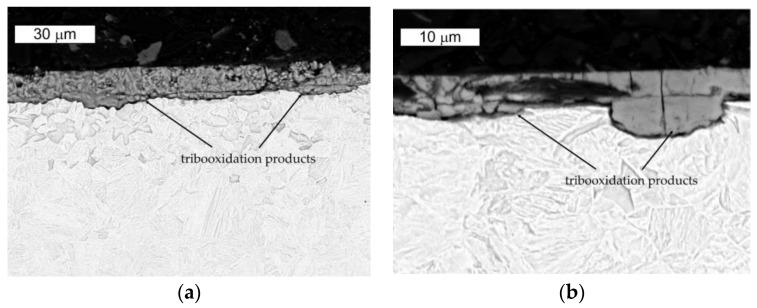
View of (**a**) the microstructure of steel Hardox in the surface area and (**b**) a magnified fragment of the area in Figure 16a in the transverse direction to the direction of the friction track. SEM, etched.

The microscopic SEM observations performed in the transverse direction to the direction of the friction examination showed the formation of surface irregularities, which point to the presence of ridging. In the formed irregularities, depositions of oxides originating from the tribooxidation process were observed. No microstructural changes in this direction were observed (Figure 16).

**Figure 17:**
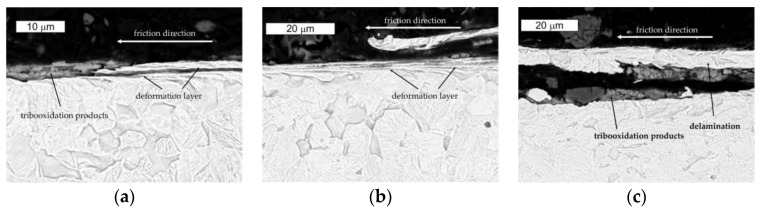
Microstructure of steel Hardox in the surface area observed in different zones. Transverse direction to the friction direction. SEM, etched.

The microscopic SEM observations performed in the longitudinal direction to the direction of the friction test showed strong plastic deformation in the microstructure area. These changes did not exceed the thickness of 10 micrometers. The delamination was simulated by the presence of numerous pressures generating high tensile stresses combined with shear stress resulting from the Hertzian contact. The weakening of the surface layer caused by these changes led to surface delamination, which was a direct cause of the creation of loose particles detached from the surface. These particles became mixed with the oxidation products. Locally, surface fragments underwent decohesion, which, on the one hand, could be connected with the formed delamination and, on the other hand, the formed oxidation products were characterized in a larger volume. This, in turn, could additionally favor the detachment of fragments of the changed surface from the matrix (Figure 17).

#### 3.8.2. Steel NC11LV

**Figure 18:**
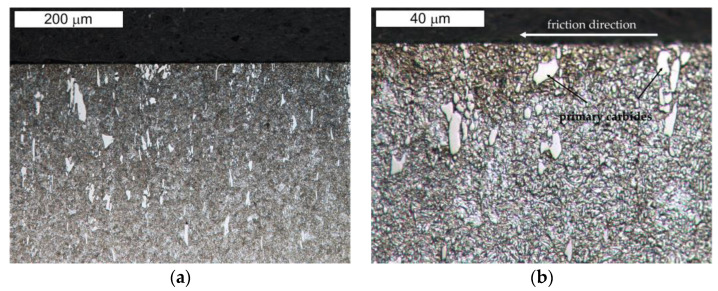
View of (**a**) the microstructure of steel NC11 in the surface area and (**b**) a magnified fragment of the area from Figure 18a. Light microscopy, etched.

**Figure 19:**
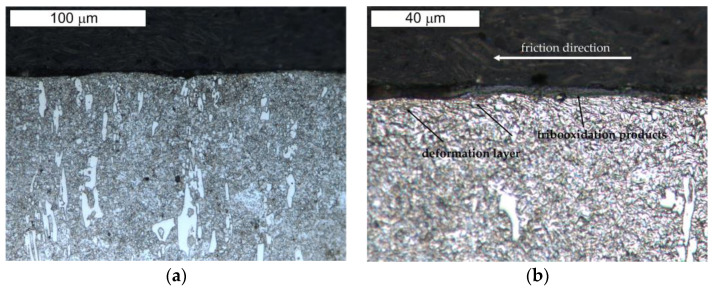
View of (**a**) the microstructure of steel NC11 in the surface area and (**b**) a magnified fragment from the area in Figure 19a. Longitudinal direction to the friction direction. Light microscopy, etched.

In the microscopic tests, no formation of spallings or material loss were observed in the surrounding of the carbide precipitates (Figure 18). Observations made in the longitudinal direction to the friction direction showed plastic deformation in the surface area (Figure 19). In the friction area, tribooxidation products were observed, which were also recorded at the stage of macroscopic tests (Figure 19b). Locally, a loss of continuity between the carbide precipitates and the matrix was observed, yet without macroscopic material decohesion.

**Figure 20:**
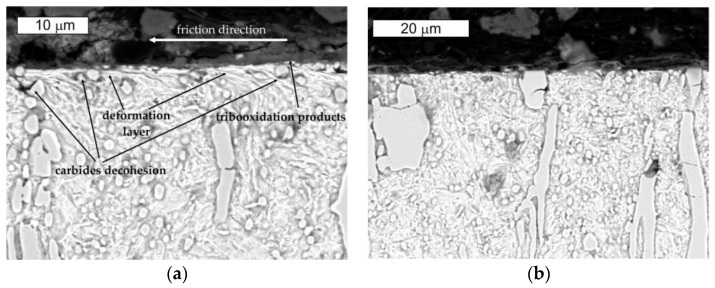
Microstructure of steel NC11LV in the surface area: (**a**) longitudinal direction to the friction direction and (**b**) transverse direction to the friction direction. SEM, etched.

The microscopic SEM observations conducted in the transverse direction to the direction of the friction test showed the formation of surface irregularities, which point to the presence of ridging. Brittle cracks of primary carbides also appeared locally, caused by surface pressures. This is due to their very high hardness. No other microstructural changes in this direction were observed (Figure 20).

The visible traces of ridging and spalling in the magnified image are the effect of a higher hardness of steel NC11LV compared to steel Hardox, where the detachment of material fractions is lower. This could also be observed in the measurements of the loss during the scanning of the friction tracks.

## 4. Conclusions

The study presents the results of investigations referring to a complex analysis of two materials, steel Hardox 600 and steel NC11LV (X153CrMoV12, Steel 1.2379, AISI D2), used for tools assigned for the extrusion of clay strands, both under industrial conditions, and in additional ball-on-disc tests. It can be concluded from the complex comparative analysis of the tools made of both steels that:

1. A higher hardness in the industrial process was demonstrated by the tools made from steel NC11LV, as they had worked about 900 operation hours, whereas the tools made from steel Hardox 600 had worked over about 400 operation hours.
2. The analysis of the geometrical loss based on the 3D scanning results showed that a higher material loss in the process was observed for steel Hardox, i.e., the average of about 0.5 mm in the normal direction.
3. The measurements of the hardness profile demonstrated that a slightly higher hardness is exhibited by the tools made from steel NC11LV, and the mean hardness equals about 61–63 HRC, with respect to about 59–60 HRC for steel Hardox 600. Even the decrease in hardness due to friction did not reduce the hardness value of NC11LV steel to the hardness of Hardox 600.
4. The wear examinations in the ball-on-disc trials showed much deeper and wider traces of wear for steel Hardox 600. The mean depths for 3 selected areas for this steel equal about 17.7 micrometers, whereas for steel NC11LV, they are about 6.6 micrometers. We can also observe slightly different characteristics of wear, i.e., for steel Hardox, it is more uniform and broader, while for steel NC11LV, we can notice certain surface irregularities, which may prove the presence of hard carbide bands.
5. The investigations of the state of the surface on samples made of steel Hardox 600 after the ball-on-disc test demonstrated that the fundamental process occurring in the friction couple was surface oxidation, which led to the formation of oxides in the friction area, causing the effect of tribooxidation. No clear differences between the two steels were observed in respect of the destruction mechanisms.
6. In the case of steel NC11LV, the character of the formed changes was similar to that of the changes taking place on steel Hardox 600, however, the intensity of oxidation was lower. Apart from tribooxidation, in both cases, ridging of the surface and slight traces of surface deformation were also recorded.
7. The presence of primary carbide precipitations does not translate to an increase in the intensity of surface destruction, as no loss of material cohesion caused by their presence at a macroscopic level, which could contribute to their being transferred into the friction couple, was observed.

On the basis of the obtained results for both the wear of the tools used to extrude strands in an industrial process and the ball-on-disc tests, it can be established that a better wear resistance (material loss) is exhibited by steel NC11LV. This justifies and predisposes the latter to be used for tools assigned for the preparation of a clay mixture strand for ceramic roof tiles, especially that steel NC11LV, in the economical aspect, is slightly cheaper than steel Hardox 600. What is more, steel NC11LV provides better possibilities to steer the properties through a change in the thermal treatment parameters, as opposed to the commercial steel Hardox supplied in one and the same state of delivery, without the possibility to determine its properties. One should also consider the aspect of production efficiency, which is connected not only with the purchase of a more expensive tool material but also with longer machine shutdowns as well as the necessity of more frequent tool replacements, which, of course, is related to lower productivity and higher unit costs of production.

Further studies will involve a subsequent optimization of bits made of steel NC11LV in the scope of thermo-chemical treatment and surface hardening, as well as other aspects. In the case of bits made of steel Hardox, protective coatings or other variants can be applied in such a way so that good parameters will make it possible to obtain the highest possible abrasion resistance.

## Figures and Tables

**Table 1:** Chemical composition of examined materials.

Element (wt.%)	C	Mn	Si	S	P	Cr	Mo	Ni	V	Cu	Fe
Hardox 600	0.44	0.98	0.17	0.004	0.009	0.094	0.051	0.69	0.04	0.01	balance
NC11LV	1.61	0.24	0.38	0.002	0.003	11.08	0.87	0.21	0.73	0.25	balance

**Table 2:** Volume measurement results.

Parameters	Hardox 600	NC11LV
Volume track (µm^3^)	9,622,503	3,397,096
Max. depth track (µm)	17.00	7.46

## Data Availability

Data sharing is not applicable.
